# You’re Fired! International Courts, Re‐contracting, and the WTO Appellate Body during the Trump Presidency

**DOI:** 10.1111/1758-5899.13032

**Published:** 2022-03-14

**Authors:** Giuseppe Zaccaria

**Affiliations:** ^1^ Department of Political Science Maastricht University Maastricht Netherlands

## Abstract

A long‐standing debate amongst international relations scholars has surrounded the question of whether international institutions with judicial authority enjoy more autonomy and discretion than other global institutions. This is mainly because international courts are established as impartial third‐party actors tasked with performing adjudicative functions for conflicting parties. As such, the delegation contracts of international institutions with judicial authority are expected to minimize control by states, even in cases where the members of a court engage in judicial overreach. This article contributes to that debate by examining the case of the crisis of the WTO Appellate Body. The article analyzes the Trump administration's successful efforts at rendering dysfunctional one of the most powerful courts in the international system. The findings showcase how powerful states are capable and willing to take advantage of the available control mechanisms and the institutional opportunity structures inherent in the design of international courts. The article speaks to the scholarship on the contestation of international institutions. The analysis relies on original data obtained through 22 interviews with WTO officials, state representatives, and experts.

**Policy Implications**

- Powerful international courts should not be viewed as immortal and shielded from state control. Protectionist, anti‐multilateralist states are willing to take advantage of opportunity structures embedded within the institutional design of such organizations to exert their influence and control on them in pursuit of their interests.
- Politicization of, and state influence on, international institutions can ultimately lead to a weakening of the authority and autonomy of these institutions. Careful designing of international institutions to ensure that states cannot take advantage of institutional opportunity structures is imperative for avoiding this.
- The Appellate Body's current dysfunctionality is very costly for the WTO, arguably threatening the future of its multilateral institutional framework for governing global trade. This dysfunctionality should not be understood as unique to the WTO but as a warning to other international legal institutions of the opportunity structures that powerful states may use to disrupt their functionality.

## 1 EXPLAINING THE CONTESTATION OF THE WTO APPELLATE BODY

The institutions at the forefront of the global multilateral order are being challenged by a wave of counter‐institutionalism, unilateralism, and bilateralism, the manifestation of which are reflected by the crisis at the World Trade Organization (WTO) (Hoekman & Mavroidis, 2021). The United States (US) has been at the center of the crisis, having consistently blocked the appointment of judges to the WTO’s Appellate Body, effectively making one of the most valuable components of the organization inoperable. To add salt to the wound, the Trump administration called the organization ‘broken’ and refrained from endorsing the nomination of Ngozi Okonio‐Iweala as the new WTO Director General, and expressed its consideration for leaving the WTO (Amaro, 2020; Hopewell, 2021). In short, the Trump administration accused the Appellate Body judges of engaging in unsolicited judicial overreach, making the case that this represents a deviation from the agreements to which member states had signed up to at the inception of the organization (USTR, 2018, 2020).

The crisis at the Appellate Body offers a relevant and puzzling case for the study of the dynamics of international delegation to international institutions with judicial authority. International courts (ICs), tribunals, and other international organizations (IOs) that have judicial authority, can be referred to as trustees. The logic of delegation to trustees is sometimes argued to be different from that of other international institutions, as the main motivation of states in establishing them is to benefit from their impartial and apolitical third‐party character (Abbott et al. 2020; Alter, 2008; Grant & Keohane, 2005; Majone, 2001). Trustee contracts are often tailored to create dispute settlement systems that are insulated from the political dynamics and pressures that are rife in inter‐state relations (Elsig & Pollack, 2014). As such, trustees may be shielded from the sort of control that states have on other IOs.

The WTO Appellate Body closely resembles a trustee. The Body is a permanent WTO division, consisting of seven judges appointed by member‐states to deliver final rulings over trade disputes. As such, the WTO Appellate Body functions as an international adjudicative court, and its mission and operations match well with those of a trustee. However, accounting for the trustee character of the Body, how can the crisis be explained? More specifically, considering that the Appellate Body is one of the most powerful and central international courts in the international system, and as such it is expected to be shielded from the political control of disgruntled states, how can its current dysfunctionality be explained?

Through an empirical investigation of the strategies employed by the US to render the Appellate Body inoperable, this article provides empirical evidence supporting the view that the delegation contracts of international institutions with judicial authority do not inherently shield them from the control of states. As the article shows, the US and other member states have for decades voiced their concerns regarding the Appellate Body through rhetorical attacks and calls for reform. However, the Trump administration took advantage of the opportunity structure built within the institution, turned words into action, and vetoed the (re)appointment of all the judges at the Body, effectively disrupting its operations. Denying the re‐appointment of staff is a classic example of a control tactic employed by states against ordinary IOs (Alter, 2008; Elsig & Pollack, 2014; Voeten, 2007). Moreover, these policies reflect wider societal cleavages and grievances within the US against the institutions of world trade, representing a backlash against the WTO and its Appellate Body.

Drawing on empirical data obtained through 22 interviews with current and former WTO and state officials, the analysis conducted here demonstrates that overtly protectionist and anti‐multilateralist administrations such as that of President Trump are capable and willing to take advantage of the institutional opportunity structures inherent in the design of international institutions by making use of the control mechanisms available to them post‐delegation. Ultimately, the case explored here highlights the enormous pressures that IOs are facing by deglobalizing actors. This is the case even for IOs with central positions and roles within the international system, such as the WTO.

In the following sections, the article delves into the International Relations (IR) literature on the international contestation of international courts, merging this with propositions from the literature on resistance/backlash against international courts in the international law scholarship. The theoretical discussion guides the article's empirical investigation, which focuses on the case of the WTO Appellate Body crisis. The article concludes with an overview of the findings and a discussion of the potential policy and academic implications, as well as the avenues for future research.

## 2 DELEGATION AND CONTROL OF INTERNATIONAL COURTS

This article argues for going beyond a discrete categorization of international organizations/courts for explaining state‐led contestation of these institutions. Even when it comes to international courts, states can take advantage of the opportunity structures inherent in the design of such institutions to block and effectively contest them, as they would with other international organizations. Furthermore, while some forms of contestation may stay limited to specific rulings through rhetorical legal and political critique of the institutions, states may employ opportunity structures to produce favorable institutional outcomes, such as rendering the institution dysfunctional without necessarily disbanding it. Finally, state‐led contestation of international courts does not only arise from divergences from the delegation contracts of such agents, but also from states’ domestic politics and the constellation of factors outside of these institutions that provide inducive conditions for state‐led challenges against them.

In other words, contextual factors (domestic politics) and institutional factors (delegation contract, institutional design) together play a role in the processes behind contestation, as well as the form it manifests itself through. These propositions build on the Principal‐Agent (P‐A) scholarship on delegation contracts of IOs but resonate as well with studies on resistance/backlash against ICs in the international law literature (due to the focus on contextual factors). The resulting theoretical framework therefore fruitfully marries insights from both bodies of research to produce an approach that converges logical conclusions regarding both contextual and institutional factors to contribute to our understanding of contestation of international institutions with judicial authority.

The accounts from the P‐A literature expect the degree of autonomy, and consequent agency, of international institutions to vary according to *ex ante* or *ex post* control mechanisms established within their delegation contract (Abbott et al. 2020; Elsig & Pollack, 2014). The former are aimed at aligning the behavior of IOs with the interests of member‐states by creating incentive structures (Abbott et al. 2020; Hawkins et al. 2006). These include rules as part of the delegation contract, institutional checks and balances, and screening and selection mechanisms. *Ex post* mechanisms, in contrast, consist of mechanisms tailored at (re)orienting the behavior of institutional actors (Abbott et al. 2020; Hawkins et al. 2006). These include monitoring and reporting (e.g., police patrols and fire alarms) and sanctioning mechanisms ranging from budgetary restrictions, re‐contracting, and in most extreme cases, closing down the institution.

In theory, all IOs face the threat of *ex ante* and *ex post* mechanisms when deviating from the rules of their delegation contract. However, it is often argued that states allow for more autonomy and discretion in the delegation contracts of ICs (Abbott et al. 2020; Alter, 2008; Alter et al. 2016; Alter & Helfer, 2010; Elsig & Pollack, 2014; Grant & Keohane, 2005). Most IOs are delegated authority with the purpose of decreasing transaction costs, are selected based on their perceived faithfulness, and their contracts are tailored to ensure a hierarchical control over them (Abbott et al. 2020; Hawkins et al. 2006; Pollack, 2003). In contrast, ICs are delegated with trustee powers to act as adjudicators in disputes, with the foremost aim of reassuring concerned parties that their interests are protected (Alter, 2008; Alter et al. 2016). In most international courts, judges are selected based on their reputation and given authority to make judgements impartially (Abbott et al. 2020; Alter et al. 2016; Elsig & Pollack, 2014).

To be able to perform those functions effectively, states desire international courts to be third‐party agents insulated from political control (Abbott et al. 2020; Alter, 2008). Competence, however, comes at the cost of control. State control would frustrate an IC’s ability to remain impartial, thus harming its credibility and competence (Abbott et al. 2020). The desire to enlist an international institution that can perform its functions with as much competence as possible (i.e. impartiality, legal and judicial professionalism, insulation from political influence) necessitates granting considerable autonomy to ICs (Abbott et al. 2020). This implies that the *ex post* mechanisms of control available to states *vis‐à‐vis* ordinary international institutions are either not feasible or rather ineffective when it comes to their relations with international courts (Abbott et al. 2020; Alter, 2008; Elsig & Pollack, 2014; Grant & Keohane, 2005; Majone, 2001).

When contestation occurs, it is expected to be mainly limited to a rhetorical challenge to the authority of the court by the contesting state(s), as compared to the more serious control and punitive tactics that states may employ against other non‐judicial IOs. Elsig and Pollack (2014) for example demonstrate that states do in fact apply certain measures against ICs that are similar to those employed against ordinary IOs. States employ various influence tactics, such as screening and selecting the personnel and judges working at an IC, to protect their interests. These, however, represent *ex ante* tactics in as much as they ensure that court staff are selected in a way as to ensure that favorable rulings are produced. In practice *ex ante* tactics are meant to offer states only with the ability to tweak the agenda‐setting process within the institution, such as by ensuring that judges with favorable views are selected. Whether such influence mechanisms may provide states with the sort of tools necessary for explicitly controlling ICs has not yet been fully explored in the P‐A literature (Abbott et al. 2020).

What is proposed here is that the delegation contract of ICs may create opportunity structures embedded within their institutional design that may allow states to employ these against the institutions despite the lack of viable *ex post* control mechanisms. More specifically, states may exert hierarchical control over those institutions through the opportunistic use of available *ex ante* mechanisms, such as appointment of staff and budgeting. Through the use of such opportunity structures, states may obtain outcomes similar to re‐contracting in their contestation of ICs. For example, by denying the (re)appointment of all judges, member states can render a court dysfunctional, effectively shutting down the institution.

Moreover, this article argues that it would be unproductive to describe ICs as a discrete type of institution. Courts in the domestic arena are distinct institutions. In contrast, on the international level the distinction between institutions with judicial authority and other types of institutions may be less clear. This is as state principals compromise and tailor the delegation contracts of institutions depending on their specific needs and the particular functions and purposes of the institutions (Abbott et al. 2020). The implication of this is that not all ICs will have the same level of autonomy or authority, and the mechanisms of state influence and control described in their delegation contracts may similarly vary.

International courts are institutions first and foremost. As recent scholarship has shown, IOs are not immortal, and many indeed perish with time (Debre & Dijkstra, 2021; Eilstrup‐Sangiovanni, 2020). Accounting for the ‘institutional’ nature of ICs, and the state‐mandated nature of their authority, it is logical to expect that the same factors and processes that lead to the decline of IOs would also play out in the case of international courts. More specifically, when international courts are deemed as deviating from their delegation contracts, powerful states with anti‐multilateralist agendas may be more likely to treat them similarly to ordinary IOs and attempt to control them.

Additionally, while institutional opportunity structures created by the delegation contract may provide the necessary tools for states to contest ICs, contextual factors also play an important role in the process leading to the contestation of such institutions. When contextual factors, specifically the domestic politics of powerful states, are inducive to anti‐multilateralist agendas and policies, the high degree of autonomy and discretion enjoyed by ICs, as well as their authority and credibility, may get directly contested. In such circumstances, powerful states may be more willing to engage not only in overt contestation against ICs, but also attempt at effectively disrupting their operations. While, at first instance, anti‐multilateralist states may resort to rhetorical attacks and playing the legitimacy politics, in the long run the sort of control tactics used against deviating IOs may become the weapons of choice against ICs as well.

The hypothesized reason behind the role played by domestic politics is that the constituencies upon which populist and anti‐multilateralist administrations rely to stay in power often include special interests’ groups that favor protectionist policies, as evidenced in the IR literature on IO contestation (Debre & Dijkstra, 2021; Eilstrup‐Sangiovanni, 2020; Heinkelmann‐Wild & Jankauskas, 2020; Voeten, 2020). When these interests are reflected in the foreign policy making process of a powerful state, it is reasonable to expect that the stance held by them becomes more aggressive, and they may become more willing to employ strong tactics against multilateral institutions, irrespective of whether these are ICs or not. The US under the Trump administration stood out in this regard, and its policies clearly reflected the anti‐multilateralist character and overt hostility of the past administration towards IOs (Heinkelmann‐Wild & Jankauskas, 2020).

The focus here on contextual factors and the process behind contestation of ICs resonates well with the propositions of the resistance approach and the wider literature on IC backlash, in particular research conducted by Caserta and Cebulak (2018), and Madsen et al. (2018). Borrowing from that approach, this article puts the spotlight on the process behind the contestation of ICs rather than the outcome. This allows us to uncover the contextual factors behind the form that state resistance against ICs can take (Madsen et al. 2018). The aim here is to shed more light on how resistance to ICs may reflect also a manifestation of political and societal cleavages and the incongruence that arises under such conditions between domestic political interests and the workings of ICs.

Resistance to ICs is often described as a situation whereby the workings of an IC are challenged (Alter et al. 2016; Caserta & Cebulak, 2018; Madsen et al. 2018). Resistance can manifest itself through a variety of forms, namely: pushback and backlash (Madsen et al. 2018). Pushback entails efforts made by relevant actors at changing the future direction of an IC through criticism and subtle forms of influence. Backlash on the other hand implies attacks on the institution aimed at achieving more radical reforms or the dismantling of the institution altogether (Madsen et al. 2018). For example, backlash occurs when a member engages in actions *vis‐à‐vis* the institution that result in the suspension of the institution's functioning abilities, such as by blocking budgets, tinkering with appointments to such a degree that would obstruct the organizational processes within the institution, or even dismantling the institution.

Backlash is observable when courts themselves (and not just their rulings) are contested by anti‐multilateralist governments representing societies in which local grievances against globalization and international institutions take center stage in domestic politics. Examples of this include cases of African regional courts, the International Criminal Court, the European Court of Human Rights, and the WTO Appellate Body (Alter et al. 2016; Caserta & Cebulak, 2018; Helfer & Showalter, 2017; Madsen et al. 2018; Nathan, 2013; Sandholtz et al. 2018; Voeten, 2020). The case of the Southern African Development Community (SADC) Tribunal is illustrative in that regard. In 2011, following a ruling by the tribunal regarding Zimbabwe's land seizures, the regional court was aggressively contested (and ultimately disbanded) under the premise that it had impinged on the sovereignty of that country. The disbandment of the court reflected the subordination of the authority of the SADC's tribunal hierarchy to the domestic political imperatives of its member states (Alter et al. 2016).

The role of contextual factors in IC contestation is also clearly reflected in the case of the US contestation of the WTO Appellate Body during the Trump administration, which is the focus of the empirical investigation in the next section. The case made here is that the crisis at the institution reflects the use of in‐built institutional opportunity structures (*ex ante* mechanisms enshrined in the delegation contract of the organization) by a powerful member‐state with an anti‐multilateralist agenda to control the court. This allowed the US to effectively achieve an outcome similar to what *ex post* control mechanisms would have accomplished against an ordinary IO, namely re‐contracting and shutting down the judicial body of the institution. These measures go beyond rhetorical and influence tactics, demonstrating the ability and willingness of powerful states to control and/or disrupt even IOs with judicial authority. Moreover, the findings resonate well with the expectations of the resistance/backlash literature, highlighting the role of domestic contextual factors in the process behind the US contestation of the WTO.

## 3 THE CASE OF THE WTO APPELLATE BODY

Since the early 2000s the US has claimed that the WTO has focused too much on its judicial functions and, in doing so, it has espoused a role that was unintended and unforeseen in the Dispute Settlement Understanding (DSU) (Interviewee #9). The Appellate Body has been accused of engaging in judicial activism, establishing a body of international trade law, and relying on legal precedence (Interviewee #1; U.S. Mission Geneva, 2019; USTR, 2018). Under the administration of President Trump these allegations came to the fore more forcefully. In 2017, the Trump administration put words into action, blocking the approval of members of the WTO’s Appellate Body, and effectively disrupting the organization's dispute settlement system (Bown & Keynes, 2020). This has resulted in an Appellate Body that, as of December 2019, lacks the required number of judges for it to function, therefore effectively spelling its demise after over two decades of operating within the institutional framework of the WTO.

The analysis demonstrates that the opportunity structures available within the institutional design of the WTO allowed for the US, a powerful member with a (at the time) distinctly anti‐multilateralist agenda, to effectively employ *ex ante* mechanisms to contest the Appellate Body in its entirety and achieve the same outcome that *ex post* control mechanisms would have achieved. As the findings demonstrate, the punitive actions by the Trump administration resulted in a significantly dysfunctional WTO and a push for a reconsideration of the organization's design, consequences that are reasonably comparable to the effects of *ex post* control tactics (re‐contracting).

The bulk of the analysis relies on the views of relevant actors within and without the organization, obtained though original interview data. In total, 22 interviews were conducted between April 2020 and February 2021. All interviews were based on the condition of full anonymity, hence quotations from interviewees are identified with anonymous labels (e.g., ‘Interviewee #1’). The majority of interviews were audio recorded and fully transcribed based on informed consent. For a descriptive list of the interviews please refer to the Appendix.

Particular attention was paid on ensuring data triangulation. The interview data draw from the views of former Appellate Body judges, as well as those of current and former WTO Secretariat staff. Interviews were also conducted with current and former state officials and representatives at the WTO, experts on international trade, as well as academics in the field of international trade law. The large number of interviews conducted aimed primarily at tracing the process behind the evolution of the US contestation against the Body in the past decade and throughout various US presidencies. Therefore, interviewees were selected based on the relevant period in which they had professional links with the organization.

Interviews were semi‐structured, with a standardized interview questionnaire guiding the process. The questions asked in the interviews were directed at obtaining the views from various actors within the organization, with the specific aim of exploring the various processes that were considered as having contributed to the Appellate Body crisis. Key questions were: ‘What role did the Trump administration play in the crisis?’; ‘How did the previous administrations differ in their approach to the Appellate Body?’; ‘What were the concerns within the Body/WTO regarding the challenge posed by the Trump administration?’; and ‘What incentive and opportunity structures within the organization shaped the Trump administration's strategy against the Body?’.

### 3.1 US contestation against WTO Appellate Body before Trump

Already in the early years of the Appellate Body's existence, powerful members such as the US and the EU relied on the (re)appointment process of judges to influence the Body. This tactic became evident during the first reappointment phase of the judges in the late 1990s, which came in tandem with the first accusations of judicial overreach against the Body. The point of contention centered on whether the DSU allowed for panels and judges to receive *Amicus Curiae* briefs, which consist of unsolicited reports and information by non‐governmental organizations (NGOs) (Elsig & Pollack, 2014; Mavroidis & Deakin, 2001). Some members argued that NGOs should not be given such special rights, and consequently brought the matter to a panel which ruled in their favor (Mavroidis & Deakin, 2001). The dispute was then brought to the Body for a final ruling. Unsurprisingly, the panel decision was overruled (Mavroidis & Deakin, 2001).

Discontenting members, particularly the EU, argued that the interpretation provided by the judges regarding the rules set by the DSU went beyond their obligations, and that the Appellate Body was trespassing its mandate by engaging in judicial overreach (Elsig & Pollack, 2014; Interviewee #9; Mavroidis & Deakin, 2001). This led to a General Council meeting in which concerned members discussed the case and attempted at overruling the Body. However, the ruling was not overturned, as this requires consensus among members (Mavroidis & Deakin, 2001).

Following that case, it became clear that attempts at overturning Appellate Body rulings were futile, as without agreement across the board, member states would be unable to react *ex post* to the judges as a collective principal (Interviewees #1, #9, #13). Consequently, members resorted to the simplest form of *ex ante* mechanism available to them for influencing the Appellate Body, namely the process of nominations and (re)appointments of judges (Elsig & Pollack, 2014). Nominations for the Appellate Body positions also call for consensus, effectively giving members veto power over appointments. This has become a frequently used method for expressing discontent with judges, with members often vetoing the reappointment of nominees that were already sitting at the court to send a clear contestation message against their previous rulings.

The next phases of (re)appointments of judges in the early 2000s followed a streak of unfavorable rulings for the US (Alter, 2008). In the decade after the establishment of the WTO, the US had lost more cases than any other member, with negative Appellate Body rulings representing a total of 70 per cent of all its appeals (Alter, 2008). In particular, the most important point of contention aggravating the relationship between the US and the Appellate Body related to the cases on antidumping practices (Hopewell, 2021; Interviewees #2, #3, #6). The US supported, and often employed, the ‘zeroing’ practice in its trade relations. This practice involves excluding transactions with a negative dumping margin when calculating weighted‐average margins of exporters’ products that are under investigation for dumping, often resulting in higher dumping margins (Schott & Jung, 2019). This trade practice by the US has led to various disputes with WTO members, resulting in cases that were taken to the WTO dispute settlement system. The US consistently lost these cases, as the Appellate Body judges regularly ruled against the zeroing practice (Interviewees #3, #6).

In response to these developments, the US began consistently and more aggressively employing *ex ante* influence tactics. The US started nominating candidates that held similar views, instead of the previous practice of offering nominees that differed in their background and legal perspectives (Elsig & Pollack, 2014). The US also blocked nominees put forward by the EU and other members whom it considered as likely to rule against it at the appeals process (Elsig & Pollack, 2014; Interviewee #9).

Additionally, the US changed its stance regarding the autonomy of the judges, and the United States Trade Representative (USTR) resorted to rhetorical attacks against rulings that it claimed reflected the over‐judicialization of the system and unsolicited judicial activism. Nominees that were perceived as favoring that growing aspect of the Appellate Body were vetoed, while those that exhibited a neutral stance were approved (Elsig & Pollack, 2014; Flett, 2010). This was also meant as a threat to other members, a clear message that unfavorable nominations would be rejected by the US. It also made it arguably clear to individual judges that their reappointment would be at jeopardy if they acted against the interests of the US (Interviewee #9). This was evidenced by the refusal of the USTR to renominate Jennifer Hillman for a second term at the Appellate Body. As argued by Elsig and Pollack (2014, p. 409), this reflected the fact that ‘the nomination process can potentially be used not only to shape the preferences of members *ex ante*, but also as an *ex post* warning to sitting members about independence from the governments that nominated them’.

The move towards influencing the incentives of the judges through the (re)appointment process came in parallel to early proposals by the US to reform the dispute settlement system by giving members more control over the process. The rhetoric that accompanied the proposals was aggressive, with the USTR declaring that the faulty decisions by the Appellate Body required counterstrategies by the US and the rest of the membership in order to ensure that the organization does not deviate from the trade agreements (Alter, 2008). This has been a consistent approach by the US throughout the two decades since the Appellate Body began functioning, relying on rhetorical attacks against the legitimacy of the judges’ rulings when these were not in its favor (Alter, 2008). The US consistently employed *ex ante* mechanisms, as evidenced by its efforts to influence the Appellate Body proceedings through the (re)appointment of individual judges (Hopewell, 2021). Nevertheless, and despite the rulings regarding the US meat and steel‐related trading practices and vocal criticisms by the USTR, the US continued to comply with the decisions (Bown & Keynes, 2020; Jung & Kang, 2004).

This period of contestation by the US shows the relational dynamic between international courts and states characterized by more autonomy for the former and less control by the latter. Although the US attempted to influence the individual court's judges through *ex ante* control tactics, ultimately the court in its entirety retained its authority and autonomy, as evidenced by the fact that the Appellate Body continued to produce rulings that were unfavorable to the US. As such, the previous paragraphs point to the lack of ability (and willingness) by the US to effectively control and rectify what was deemed as a deviating international court. However, as the next subsection illustrates, the US–WTO relations changed dramatically during the presidency of Donald J. Trump, which reflected not only the contrast in policies by a different administration but also an altered political domestic scene in the US.

### 3.2 US contestation against WTO Appellate Body during Trump

Even before becoming US President, Donald Trump made it very clear that he intended to bellicosely go after the WTO and its dispute settlement system. The first signs of this approach became evident early on in his presidency. Trump claimed that the WTO and the Appellate Body are a challenge to his ‘America First’ policies, making it impossible for the US to effectively obtain fair trade terms with its international partners and inhibiting its ability to protect its domestic market, workers, and large industries (Swanson, 2019). Furthermore, Trump, the USTR Lighthizer, and other actors within the trade community in the US consistently accused the organization and the Appellate Body of not taking China's trade violations seriously (Interviewees #1, #9, #13).

The core of the criticism involved the decision by the WTO to allow China to hold the status of a developing country, which the US considered as unfair given that the country's economy is the second largest globally (Interviewee #9; Mavroidis & Sapir, 2021). This has allowed China to subsidize its domestic products, giving it a competitive edge in its trade relations with other WTO members. At the same time, China and other members have received favorable rulings on disputes regarding the use of tariffs by the US on foreign products. This, the Trump administration argued, had emboldened China to continue its policies of state subsidization of private enterprises and the protectionist measures provided for exporting industries, allowing its economy to benefit disproportionately at the expense of American industries (Interviewee #9). These concerns were shared by members of Congress and various industry representatives, reflecting their relevance in the domestic context within the US (Interviewees #1, #9).

In his first year in office, President Trump and his administration immediately began devising trade plans that deviated from the rules of the WTO. The rhetorical attacks against the organization grew fiercer, but they were also accompanied by threats of imposing tariffs on trade partners if the WTO did not take action against trade violations by China. This would require the consensus of WTO members, thus impeding an effective response by the organization, and allowing the Trump administration to make accusations of complacency and ineffectiveness to impose fair trade practices among its membership (Interviewee #1).

In response to the claimed inability of the WTO to counter alleged malpractices by China in the area of trade, the US unilaterally imposed tariffs on Chinese products in an effort to protect its domestic producers and gain a political leverage to obtain better trade terms in bilateral negotiations. The Trump administration also applied tariffs against other WTO members, such as the EU, Turkey, Japan, and Brazil, sidestepping the regulatory framework established under the WTO (Josephs, 2019; Swanson, 2019). This was in clear contrast to the previous Clinton and G.W. Bush administrations, which consistently remained compliant with Appellate Body rulings regarding steel and meat import tariffs (Alter, 2008). In fact, the US remained compliant with the generally unfavorable rulings produced from the nearly 90 cases brought against it by 2017 at the Appellate Body revolving around issues with safeguards, antidumping practices, and countervailing measures (Bown & Keynes, 2020). The Trump administration, however, made it clear that the US industries’ interests trumped the legal interpretations of the judges, evidencing once again the role played by domestic contextual factors in its policies against the organization.

The threat to impose tariffs came in tandem with a full‐on rhetorical assault against the Appellate Body of the WTO. The Trump administration threatened to block the (re)appointment of all of the court's judges. When proposals for reform of the Body were pushed by other members, the US claimed that these did not address its concerns satisfactorily and thus refused to compromise. While the threat of blocking the entire appeals’ process continued, US officials, and in particular the USTR, continuously reaffirmed their belief that the Appellate Body was flawed, had too much autonomy, and its judges had engaged in judicial activism for too long, with the US ambassador to WTO Dennis Shea stating at the WTO General Council meeting in July 2019 that ‘the Appellate Body has felt free to depart from what members agreed to’ (U.S. Mission Geneva, 2019).

In fact, the Appellate Body represents a pocket of autonomy within the WTO, with the division functioning completely autonomously from the rest of the organization (Interviewee #6, #7). This extraordinary delegation of autonomy was intentional in as much as it reflected the members’ interest in ensuring that the Body and its staff remain insulated from the rest of the organization and guaranteeing their impartiality (Interviewee #6). In fact, Appellate Body reports are adopted through negative consensus, making attempts at authoritative re‐interpretations of Appellate Body reports, and in effect controlling the Body, unfeasible.

However, US officials argued that despite its autonomy, the Body was still expected to stick to the rules, and the alleged procedural violations, the judicial overreach, and the unsolicited legal interpretations based on case precedence were unforeseen by the members at the time of the institution's inception, representing a trespassing of authority by the Appellate Body (Interviewees #7, #13). One interviewee pointed out that the US simply had not predicted that the organization would evolve and become a ‘different animal’ (Interviewee #14).

More importantly, the high degree of autonomy of the Appellate Body meant that influencing the nomination and (re)appointment process of judges did not necessarily imply control over the Body. This realization may have been central to the Trump administration's decision to block the entire appeals’ process. Blocking the appointment and reappointment of individual judges had been a practice for a while (Hopewell, 2021). In fact, the Obama administration also blocked the reappointment of the Korean ABM nominee Seung Wha Chang back in 2016 (Hopewell, 2021). President Obama also refused to renominate Jennifer Hillman after her first term.

Under the Trump administration, the US took this practice onto a new level. Under the claim that the Appellate Body had failed at generating fair rulings and to follow the framework provided by the trade agreements reached under the auspices of the institution, the Trump administration resorted to something that no other member had done before, namely continuously blocking the appointment and reappointment of all judges and nominees. President Trump also threatened to block the budget of the Appellate Body and stated that ‘if they [the WTO] don't shape up, I would withdraw from the WTO’ (Beatie, 2019; Micklethwait et al. 2018, p. 1).

Trump's attacks on the Appellate Body effectively harmed the organization itself and reflected a threat to the rules‐based international and multilateral order on matters of trade (Hopewell, 2021). Without a functioning Appellate Body, one of the three pillars of the WTO has essentially fallen apart, making the organization less central and posing an existential threat to the entire institutional framework.

It is clear that the past US administration completely disregarded the trustee nature of the Appellate Body, thus viewing the Body as an agent that needed punishment for deviating from the rules. This is evidenced by the discrepancy with which the US treated the Appellate Body. In fact, while the Trump administration praised the Body when it produced rulings that were favorable to the interests of the US, the opposite occured when the rulings went against its interests (Bown & Keynes, 2020; Josephs, 2019; Swanson, 2019). The Trump administration instead relied on unilateralism and bilateralism to protect its interests, criticizing the Body and questioning its credibility and impartiality (Hopewell, 2021; Josephs, 2019; Swanson, 2019).

Furthermore, the act of blocking the entire Appellate Body represented a disconnect from past US administrations. This tactic reflected the aggressive use of *ex ante* control mechanisms for effectively pushing an international court into a breaking point so as to force it to reform or essentially cease its operations. This is similar to the function and expected outcome of re‐contracting. As one interviewee noted, Trump's logic behind blocking the appointment of judges was to gain enough leverage to force the institution to rectify its behavior and follow the demands of the US (Interviewee #5).

In other words, the Trump administration's actions reflected its strategy for reining in on what it considered as a deviating agent that had become harmful to the domestic interests of the US. The imposition of tariffs on imported goods was a tactic employed by the Trump administration to redress the trade imbalance which was claimed to have been caused by the faulty process of the DSB (Interviewee #3). However, the developments at the Appellate Body were a proper case of full‐on punitive strategies being employed by a disgruntled and powerful state against an international court that was being treated as an ordinary non‐judicial institution.

Whereas there may have been a disconnect between the Trump administration's policies and those of its predecessors towards the Body, they were nevertheless in line with broader societal cleavages present within the US. In fact, while the US electorate in the decade preceding the Trump administration overwhelmingly expressed support for IOs such as the WTO, the US public's view has shifted significantly since then (Kim & Durkin, 2020; McGuinness, 2009). In fact, throughout the Trump's tenure, its administration's policies on world trade and the WTO were supported by the wider public in the US (Interviewee #1; Kim & Durkin, 2020).

The negative views on the WTO and the global institutional framework behind world trade are in line with the views of US trade officials in the United States (Interviewee #1). Recently there has also been bipartisan alignment in the US Congress regarding the WTO, reflected by the fact that, as of May 2020, both the House of Representatives and the Senate had resolutions and proposals introduced at their sessions proposing the US withdrawal from the WTO (Levy, 2020).

The overall domestic support for the Trump administration's policies against the WTO Appellate Body therefore reflected a backlash by the US public against the organization and its institutional framework. Since his inauguration, President Joe Biden and his administration appear to be keeping the same line of policies against the WTO. As of yet no remarks have been made by the Biden administration regarding a change in direction or proposals for reforming the institution. This represents continuity in the country's stance towards the Appellate Body despite the public support for multilateralism expressed by President Biden (Howse, 2021).

In sum, in previous instances of contestation of the WTO by the US, there was a clear degree of discontent exhibited by the US, albeit this was expressed through rhetorical and, in the more extreme cases, *ex ante* influence tactics. In this more recent instance, however, it is clear that the US under the Trump administration changed tactics and applied serious punitive measures against the Appellate Body and the WTO as a whole, with effects that were very similar to the *ex post* control tactic of re‐contracting. Those tactics went beyond the rhetorical and legitimacy politics employed previously by the US and represented the use of opportunity structures within the design of the international institution.

As the case study illustrates, powerful states with anti‐multilateralist agendas possessing the necessary political clout and means to engage in substantial and direct forms of contestation and institutional control, can and will take such direction *vis‐à‐vis* international courts that are deemed as harming their interests. This is reflected in a statement by the former chairman of the Appellate Body, James Bacchus, saying that there is very ‘little chance of resolving this [crisis at the Appellate Body] while Donald Trump is still president in a way that will continue to preserve the independence and impartiality of the Appellate Body and the rest of the WTO dispute settlement system’ (Josephs, 2019).

The administration's policies reflected the relevance of the shifting domestic context within the US, and how resistance to international institutions by societal and special interests’ actors within the US was manifested through the case of the demise of the Body. Whether the new administration will change tactics *vis‐à‐vis* the Body is yet to be seen, however the analysis highlights the fact that such change would be conditional on the domestic context within the US.

## 4 CONCLUSION

This article has focused on the Trump administration's successful efforts at rendering the WTO Appellate Body inoperable. The findings showcased how powerful states are capable and willing to take advantage of the opportunity structures inherent to the design of international judicial institutions to control them. During the administrations of George W. Bush and Barack Obama, the US expressed its discontent with the Appellate Body through rhetorical attacks and by employing *ex ante* mechanisms to influence the appointment of individual judges at the court. However, the Trump administration employed the same mechanisms to deny the appointment of all the judges at the Appellate Body, therefore rendering the entire court inoperable and resulting in its effective shutdown. This tactic represented the use of existing opportunity structures within the design of the institution with effects that were very similar to the *ex post* control tactic of re‐contracting.

The conclusion reached here is that describing international judicial institutions as being generally shielded from the control of states due to the nature of their delegation contract is not a productive approach for examining the influence of states on such institutions. With a protectionist and anti‐multilateralist administration such as that of President Trump, an international body with judicial authority such as the Appellate Body may receive the same treatment as an ordinary international institution when deemed to be violating the rules of the game. While powerful states may resort to rhetorical attacks and playing the legitimacy politics at first instance, in the long run the sort of tactics available to them for controlling international institutions may become the weapons of choice against international courts as well.

The consequences of the previous US administration's policies have already become evident. The current dysfunctionality of the Appellate Body is very costly for the WTO, arguably putting at risk the future of its multilateral institutional framework for governing global trade (Hoekman & Mavroidis, 2021; Hopewell, 2021; for a contending voice, see Vidigal, 2019). The demise of the Body risks ‘corroding the rules‐based trading system’ and thus represents at its core ‘an existential threat to the WTO’ as an institution (Schott & Jung, 2019). The crisis not only threatens to wane the compliance of member states and the enforcement of obligations to the organization's regulatory framework, but it also seriously undercuts the hopes for future negotiations at the WTO (Hoekman & Mavroidis, 2021). Signs of this threat have already surfaced, with various members proposing an alternative institutional framework for interstate trade‐related appeals (Howse, 2021). Whether this represents a challenge to the centrality of the WTO in the global trading system is yet to be seen.

The case explored here highlights the enormous pressures that IOs are facing by deglobalizing actors such as the US and the effects of resistance and backlash against their institutional frameworks. This is the case even for IOs with central positions and roles within the international system. Powerful international courts are not immortal and shielded from state control. Protectionist, anti‐multilateralist states are a threat to the global liberal order and its institutions, and they are willing to take advantage of opportunity structures embedded within the institutional design of such organizations to exert their influence and control.

In that regard, institutional reforms at the WTO are critical to the survival of multilateralism and the liberal international trade system (Howse, 2021). Politicization and state counter‐institutionalization could in the long‐term lead to a deepening of global governance if they are met with substantial institutional reforms that accommodate for changing global environments and power shifts in the international system (Zürn, 2018). However, without such reforms, counter‐institutionalization and state contestation of international institutions could lead to gridlock and a gradual dismantling of the institutional framework behind the liberal international order (Debre & Dijkstra, 2021; Hale et al. 2013; Zürn, 2018).

The findings of this article are therefore relevant to the broader IR research agenda on the modes and tactics of contestation of international institutions, as well as the discussion regarding the current challenges to the liberal international order and resistance to international judicial institutions. The IR literature on IOs may benefit from more research on the process of contestation of such institutions, and more focus on the institutional responses of IOs to these challenges could help uncover the role played by institutional actors in ensuring the adaptiveness and resilience of their institutions.

## 1 LIST OF INTERVIEWS

**Table:**

*Interviewee #*	*Interview date*	*Interviewee's position*
(1)	20/03/2020	International trade expert
(2)	24/03/2020	EU official
(3)	25/03/2020	Former WTO official
(4)	01/04/2020	WTO Senior Counsellor
(5)	02/04/2020	Former Appellate Body judge
(6)	03/04/2020	EU official
(7)	06/04/2020	Former WTO General Council official
(8)	07/04/2020	Former Appellate Body judge
(9)	08/04/2020	Former Appellate Body official
(10)	04/06/2020	WTO Dispute Settlement attorney
(11)	05/06/2020	EU official
(12)	09/06/2020	Former WTO official
(13)	16/06/2020	Former WTO Dispute Settlement lawyer
(14)	22/06/2020	Former Appellate Body judge
(15)	23/06/2020	Former Appellate Body judge
(16)	29/06/2020	Former WTO official
(17)	16/02/2021	Former WTO official
(13b)	17/02/2021	Former WTO Dispute Settlement lawyer
(11b)	18/02/2021	EU official
(12b)	23/02/2021	Former WTO official
(16b)	24/02/2021	Former WTO official
(18)	26/02/2021	Former Appellate Body official
